# Understanding caregiver burden with accessing sickle cell care in the Midwest and their perspective on telemedicine

**DOI:** 10.1186/s12913-023-09383-x

**Published:** 2023-05-17

**Authors:** Seethal A. Jacob, Jillian Bouck, Roua Daas, Meghan Drayton Jackson, Julia E. LaMotte, Aaron E. Carroll

**Affiliations:** 1 Center for Pediatric and Adolescent Comparative Effectiveness Research, Indiana University, 410 W. 10th Street, Suite 2000A, Indianapolis, IN 46202 USA; 2 Division of Pediatric Hematology Oncology, Department of Pediatrics, Riley Hospital for Children, Indianapolis, IN 46202 USA; 3 Indiana University School of Medicine, Indianapolis, IN 46202 USA

**Keywords:** Telemedicine, Telehealth, Sickle cell disease, Pediatric, Access to Health Care

## Abstract

**Background:**

Survival for children with sickle cell disease (SCD) has improved significantly. However, patients with SCD still encounter several impediments to accessing adequate healthcare. Rural and medically underserved areas, such as parts of the Midwest, can exacerbate these barriers, separating children with SCD from subspecialists even further. Telemedicine has been a means to close these gaps in care for children with other special healthcare needs, but few studies have discussed how caregivers of children with SCD perceive its use.

**Methods:**

The objective of this study is to understand the experiences of caregivers of pediatric SCD patients in a geographically diverse area in the Midwest in accessing care, and their perspectives of telemedicine. Caregivers of children with SCD completed an 88-item survey via a secured REDCap link either in-person or via secure text. Descriptive statistics (means, medians, ranges, frequencies) were performed for all responses. Univariate chi square tests were performed to analyze associations, particularly with telemedicine responses.

**Results:**

The survey was completed by 101 caregivers. Nearly 20% of families traveled more than 1 hour to reach the comprehensive SCD center. Other than their SCD provider, caregivers reported their child having at least 2 other healthcare providers. Most barriers caregivers identified were financial or resource based. Almost a quarter of caregivers expressed feeling as though these barriers impacted their and/or their child’s mental health. Ease of access to team members, as well as scheduling, were common facilitators of care cited by caregivers. The majority were willing to participate in telemedicine visits, regardless of how far they lived from the SCD center, though many noted aspects requiring adaptation.

**Conclusion:**

This cross-sectional study describes barriers to care experienced by caregivers of children with SCD, regardless of proximity to an SCD center, as well as caregiver perceptions of the usefulness and acceptability of telemedicine for SCD care.

**Supplementary Information:**

The online version contains supplementary material available at 10.1186/s12913-023-09383-x.

## Introduction

Sickle cell disease (SCD) is an inherited hematologic condition that affects approximately 100,000 individuals in the United States and carries with it potentially devastating complications. However, improved screening and preventative measures have led to increased survival to adulthood for children with SCD in the U.S.[1] Evidence-based, comprehensive SCD care has been shown to improve patient outcomes, but barriers to such care are well-described for children, as well as adults living with SCD.[2].

Children with SCD have also been shown to experience difficulty with transportation, extended wait times, and an inability to access subspecialty providers easily.[3] This has resulted in less than 70% of children with SCD receiving recommended clinical care, and fewer than half experiencing coordination of care between primary and subspecialty providers.[4] Further complicating issues is the fact that comprehensive sickle cell centers are most commonly located in major metropolitan areas, making it difficult for families in rural or medically underserved areas to access this type of comprehensive care.[5, 6].

Caregivers of children with special healthcare needs, such as SCD, also experience significant burdens. The physical, psychological, and financial stressors associated with caring for a child with a chronic disease contributes to this. Studies have shown that the amount of time caregivers spend each day to meet their child’s needs, as well as what they spend their time doing (e.g. physical care, supervision, etc.), introduce risks to their overall well-being.[7] Importantly, the psychological impact of this leads to symptoms of depression and anxiety, low levels of resilience, and poor family functioning.[8, 9].

Telemedicine, the delivery of healthcare from a distance with the use of audio and visual technology, has been an effective means of increasing access to subspecialty care, especially for those with chronic health conditions. Two main forms of telemedicine include: (1) Hub-and-spoke, in which an expert or specialist at a tertiary care center (hub) provides medical care virtually for a patient located at a remote clinic site (spoke), and (2) Direct-to-consumer, in which the expert or specialist provides virtual medical care directly to the patient who is often utilizing a smart phone or tablet in their home. Importantly, the feasibility of telemedicine in pediatric populations has been established, particularly during the recent COVID-19 pandemic.[10–12] Yet, there are still several gaps in the existing literature, barriers to accessing care specifically for pediatric patients with SCD have not been widely published, and little is known about how caregivers and patients with SCD feel regarding the use of technology like telemedicine for addressing these barriers [13].

The purpose of this study was to increase understanding of the experiences caregivers of children with SCD throughout a geographically diverse area of the Midwest have in accessing SCD care, as well as discern the caregiver perspective on the use of telemedicine for delivering comprehensive care.

## Methods

### Participants/procedure

Caregivers of patients with SCD seen at Riley Hospital for Children in Indianapolis, Indiana were contacted by the research team in-person or via secure text. The Riley SCD program serves patients throughout the state of Indiana, as well as parts of Kentucky and Illinois, all of whom were eligible for this survey. The primary location (i.e. hub) of the program is in Indianapolis, IN, which is in the center of the state. Satellite clinics and/or spoke telemedicine sites assist with providing specialized care in two additional cities, though patient care needs and navigation are streamlined through the hub in Indianapolis. Surveys were completed independently by caregivers via a secure Research Electronic Data Capture (REDCap) link. For surveys administered in-person, the research team was made available to provide clarification or aid with technological difficulties. Participants were compensated with a ten-dollar gift card for their time. This study was approved by the Indiana University Institutional Review Board (IRB).

### Measure and analysis

An 88-item survey was developed based on previously performed semi-structured interviews with caregivers of children with SCD and the validated Barriers to Care Questionnaire (BCQ).[13, 14] The survey included questions regarding their experience accessing care, including barriers and facilitators of care, as well as their perspective on the use of telemedicine in SCD. Descriptive statistics (means, medians, ranges and frequencies) were performed for all responses to survey questions. Univariate chi-square tests were used to analyze possible associations between numbers of barriers reported and time (as a categorical variable) to SCD center, as well as between willingness to participate in telemedicine and prior telemedicine use, perceived positives/negatives of telemedicine, caregiver worry about others caring for their child, and time to SCD center.

## Results

### Participants

**Table 1:** Baseline Characteristics of Caregivers and Children (N = 101)

	Caregivers	Children
Race/Ethnicity (select all that apply)		
African American or Black African or Black Caribbean or Creole White Multiracial Hispanic/Latinx	68% (n = 69) 22% (n = 22) 4% (n = 4) 8% (n = 8) 1% (n = 1) 0% (n = 0)	78% (n = 79) 22% (n = 22) 2% (n = 2) 1% (n = 1) 1% (n = 1) 1% (n = 1)
Relation to Child with SCD		
Birth mother Birth father Adoptive mother Adoptive father Grandmother/Grandfather Other family member Protective guardian Other	81% (n = 82) 9% (n = 9) 3% (n = 3) 0% (n = 0) 2% (n = 2) 1% (n = 1) 3% (n = 3) 1% (n = 1)	- - - - - - - -
Number of Children with SCD		
1 child 2 children 3 children 4 or more children	82% (n = 83) 15% (n = 15) 1% (n = 1) 1% (n = 1)	- - - -
Child’s Sickle Cell Genotype		
SS SC SBeta + Thalassemia SBeta 0 Thalassemia I don’t know	- - - - -	59% (n = 60) 29% (n = 29) 5% (n = 5) 2% (n = 2) 6% (n = 6)

Of the 300 caregivers contacted, 101 completed the survey, resulting in a response rate of 34%. The majority had 1 child with SCD (range 1 to 4), with most having Hb SS genotype (n = 60) (Table 1). Most caregivers identified as the birth parent (n = 91), and 39% said they had no one else to share caregiving responsibilities with. 18% of families drove more than 1 hour to access comprehensive sickle cell care.

### Caregiver Burden

Caregivers reported having a median of 2 other healthcare providers that their child sees regularly. However, nearly 10% did not have an established primary care provider. Only 10% reported being uninsured themselves, and 3% reported their child being uninsured at the time of the survey, though 38% reported gaps in insurance for either themselves or their child at some point. Nearly 13% felt as though insurance, or the lack thereof, had prevented their child from receiving the care they needed at any point in time. 17% reported receiving financial support from their healthcare team to pay bills, reimburse gas, or purchase food.

42% of caregivers who identified as the birth parent reported not knowing they carried sickle cell trait prior to their child being born. Approximately 40% felt having a child with sickle cell disease impacted their daily stress and/or mental health. 63% of caregivers worried about others (e.g. school staff) caring for their child, and nearly 50% worried about the care and knowledge of medical providers other than their SCD team. 10% of caregivers reported their child not receiving the care they needed in the emergency department at some point in time, 8% reporting this concern when their child was hospitalized, and only 2% when seen in sickle cell clinic (Supplemental Table).

### Barriers/Facilitators

**Fig. 1:**
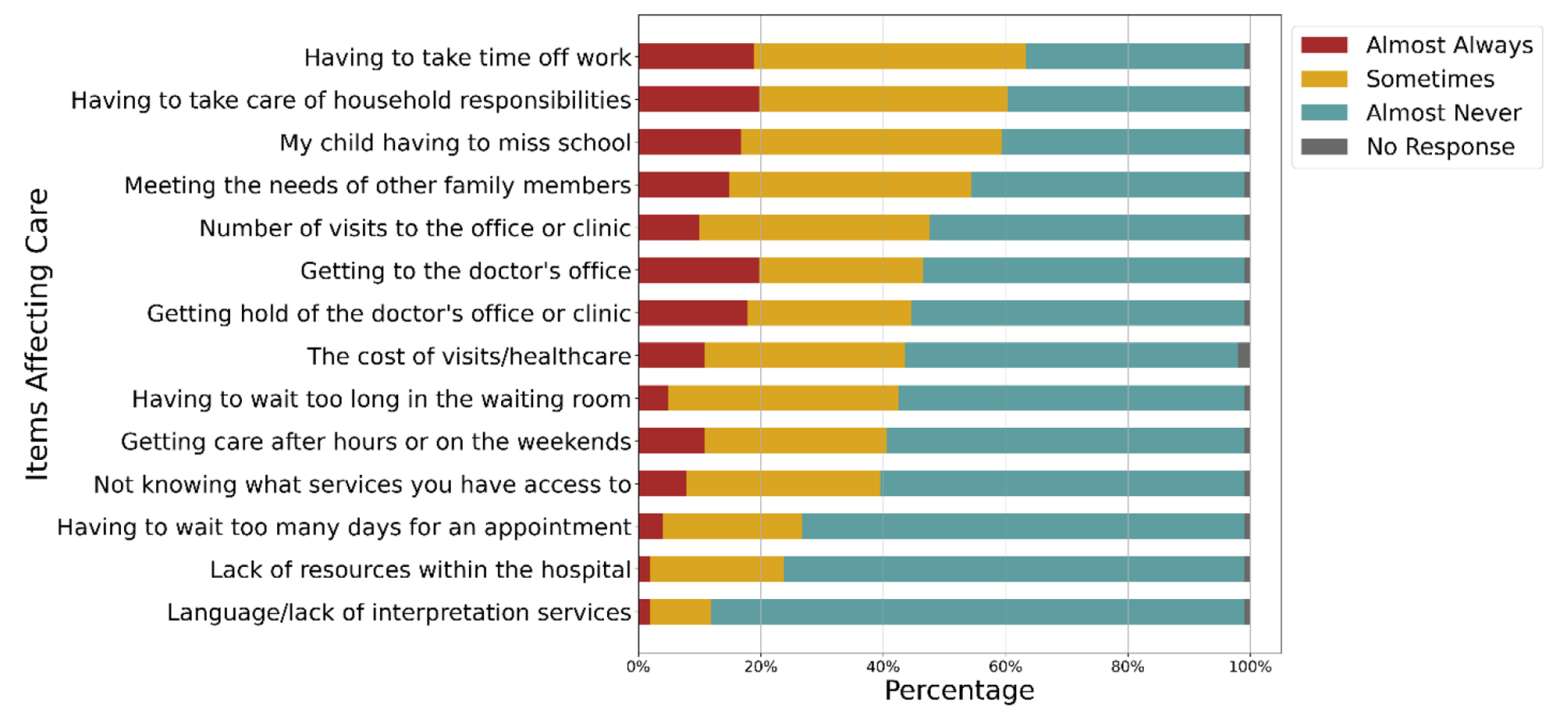
Items that affect ability to access medical care (N = 101)

Caregivers identified a median number of 6 barriers to accessing SCD care, with other household responsibilities, childcare, lost time from work and school as the most common (Fig. 1). There was no statistically significant association between travel time to the clinic and the number of barriers identified (*p* = 0.1). Fifty-two caregivers reported being the primary caregiver for someone other than their child with SCD. About 20% of caregivers felt the barriers they experienced affected their and/or their child’s well-being and/or mental health at times, yet less than half reported their healthcare team ever discussing ways to address these barriers.

Sixty-two caregivers reported sharing caregiving responsibilities with at least one other person, with nearly 70% (n = 69) stating they receive support from their family when needed. The majority of caregivers felt that it was always or sometimes true that the SCD clinic facilitated care by easy access to SCD staff, ease of scheduling appointments, and convenient times to schedule. Additionally, 17% of caregivers reported the SCD clinic/hospital supporting them through resources, with mental health support, food pantry, and housing assistance being the most common.

### Telemedicine

**Fig. 2:**
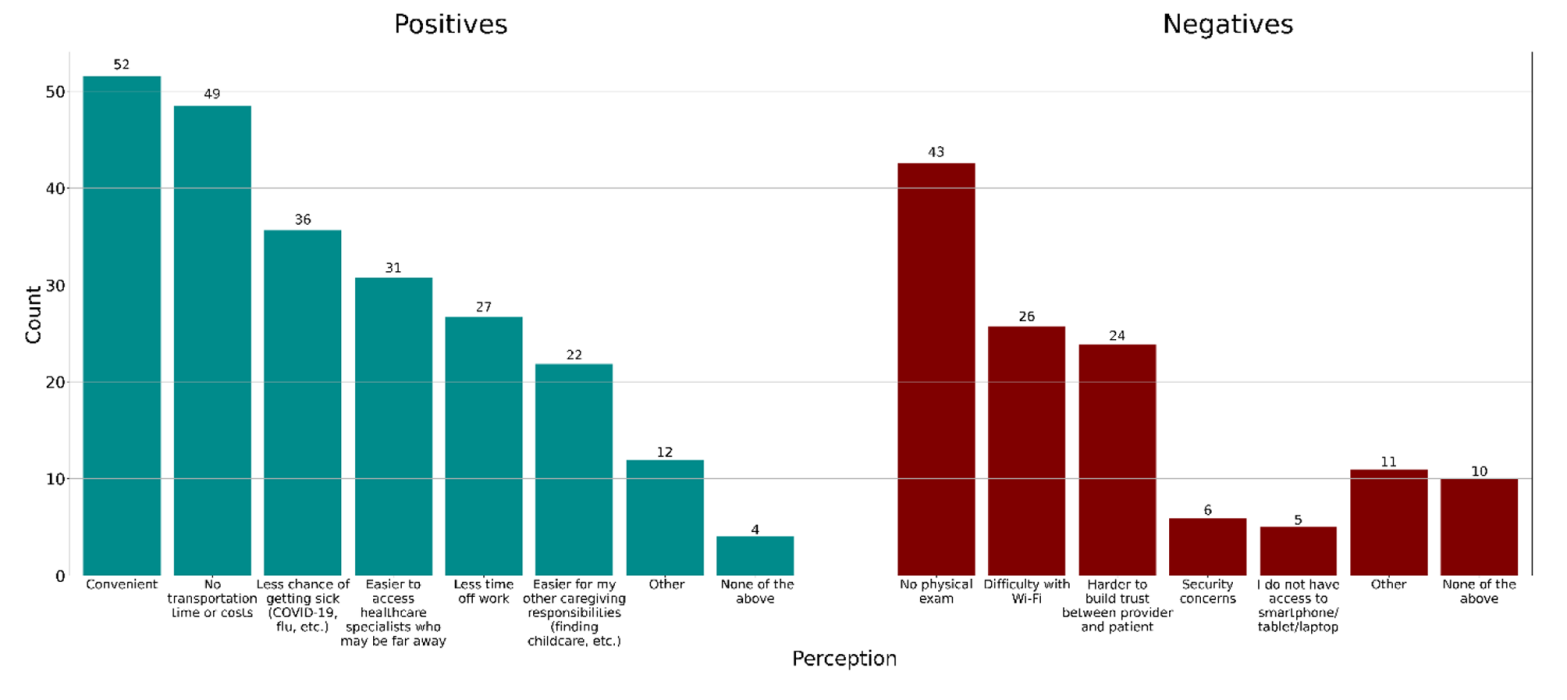
Perceived positives and negatives of telemedicine (N = 101)

40% of caregivers had participated in some form of telemedicine, the majority being direct-to-consumer. The most common positives of telemedicine reported were (1) convenience; (2) decreased transportation time/cost; (3) less chance of getting sick ; and (4) easier to access healthcare specialists (Fig. 2). However, nearly half of all respondents felt the lack of a physical exam was a negative for telemedicine, and about 25% (n = 24) stated it was harder to build trust via telemedicine. 81% (n = 82) were willing to participate in future telemedicine visits sometimes or always (Supplemental Table). This was similar for both SCD and other medical visits. There was no statistically significant association between willingness to participate in telemedicine and prior telemedicine use, perceived positives/negatives of telemedicine, caregiver worry about others caring for their child, or distance from SCD center (univariate analyses; all *p* > 0.05).

## Discussion

Access to care is a known challenge for patients with SCD, particularly because of a lack of comprehensive SCD treatment centers throughout the country. This study describes the perspectives of caregivers of children and adolescents with SCD, regardless of distance from an SCD center, in a Midwestern state with significant variation in medical resources.

While our study demonstrated similar barriers to those reported in other studies such as transportation and lost time from school and work, [15–17], the frequency of barriers reported varied, which suggests the importance of screening for barriers universally in SCD as needs may differ based on state/region. Additionally, participants in our study did report unique barriers not described in previous studies with caregivers, specifically regarding provider knowledge and bias leading to children with SCD not receiving the care they need. We had previously identified this in semi-structured interviews with caregivers who lived in rural or medically underserved areas far from the SCD center[13]. However, this concern was consistent amongst caregivers regardless of distance to the SCD center and supported by prior studies demonstrating lack of provider knowledge and comfort, as well as bias, leading to inequities in care delivery such as poor adherence to stroke screening, pneumococcal vaccination, and emergency department care [4, 18–23]. This highlights the importance of improved education and awareness regarding SCD and pain management, as well as bias, for medical providers.

Our statewide survey also demonstrated significant caregiver burden for those with a child with SCD. Caregivers reported a median of 6 barriers to accessing care and believed this impacted their and/or their child’s well-being and mental health. While it is well-known that caregivers of children with chronic diseases have increased stress, as well as increased mental health concerns, access to support for both remains limited in SCD.[8, 9, 24, 25] The implications of this are significant when considering how caregiver physical and mental health directly impact the health of the child and are linked with adverse outcomes for both.[26].

There is little literature expanding on what caregivers and/or patients believe are facilitators to *accessing* care for SCD, as much of what we consider to be facilitators is extrapolated from known or reported barriers. However, studies related to asthma care and mental health cite coordination of care, flexibility in scheduling, and addressing resource barriers as facilitators of care.[27, 28] In our study, caregivers identified several facilitators to accessing care, including accessibility of dedicated SCD team members, such as a nurse coordinator, as well as co-location of care and consolidation of appointments. Additional facilitators included the healthcare team assisting with resources, again pointing to the importance of screening universally for social determinants of health.

The COVID-19 pandemic led to increased adoption of telemedicine, specifically, the direct-to-consumer model. As a result, a significant percentage of caregivers who completed the survey had some experience with telemedicine. And while the majority of participants reported a willingness to participate in future telemedicine visits, many cited the concern for a lack of a physical exam with the direct-to-consumer model. Additionally, worries regarding building trust with healthcare team members that may be new to them was also one of the most common concerns raised by caregivers who had experienced direct-to-consumer telemedicine for both sickle cell disease visits, as well as other subspecialty visits. A small number of caregivers had participated in the hub-and-spoke model of telemedicine, but those that did, did not cite these concerns, which is consistent with the results of our previous qualitative work.[13].

Limitations to the study include the survey being limited to one state and institution. Though, Riley is the largest pediatric sickle cell center in the state and its catchment area is broad, therefore, representing a regionally diverse (rural, urban, suburban) population. Additionally, the response rate was 34%, and therefore, there may be variations or statistical significance that could not be ascertained with this response size.

## Conclusion

The experiences of caregivers accessing healthcare for their child with SCD demonstrates significant caregiver burden and barriers to care regardless of nearness or accessibility of medical care. Additionally, while the majority are willing to participate in telemedicine for future sickle cell care, the negatives of telemedicine that caregivers reported are significant. Future studies should evaluate models of telemedicine care adapted for this specific population.

## Electronic supplementary material

Below is the link to the electronic supplementary material.

Supplementary Material 1

## Data Availability

All data generated or analyzed during this study are included in this published article and its additional files.

## Abbreviations

SCD: Sickle Cell Disease
REDCap: Research Electronic Data Capture
IRB: Institutional Review Board
BCQ: Barriers to Care Questionnaire
