# Supplementary material for: Understanding caregiver burden with accessing sickle cell care in the Midwest and their perspective on telemedicine
Source: BMC Health Serv Res. 2023 May 17;23:500. doi: 10.1186/s12913-023-09383-x (PMC10189684; doi:10.1186/s12913-023-09383-x)
Supplement: Supplementary file 1 — Supplementary Material 1 [file 12913_2023_9383_MOESM1_ESM.docx]

| Supplemental Attachment – Telemedicine Survey (N=101, unless otherwise indicated) | | |
| --- | --- | --- |
| **General Questions** | | |
| 1. How many children with SCD do you have?  1 child  2 children  3 children  4 or more children  No response given | 82% (n=83)  15% (n=15)  1% (n=1)  1% (n=1)  1% (n=1) | |
| 2. Your [caregiver’s] race and ethnicity (select all that apply)  African American or Black  African or Black  Caribbean or Creole  White  Multiracial | 68% (n=69)  22% (n=22)  4% (n=4)  8% (n=8)  1% (n=1) | |
| 3. How are you related to your child with SCD?  Birth mother  Birth father  Adoptive mother  Adoptive father  Grandmother/Grandfather  Other family member  Protective guardian  Other | 81% (n=82)  9% (n=9)  3% (n=3)  0% (n=0)  2% (n=2)  1% (n=1)  3% (n=3)  1% (n=1) | |
| 4a. IF ONLY 1 CHILD WITH SCD: what type of SCD does your child have? (N=83)  SS  SC  SBeta+ thalassemia  SBeta0 thalassemia  I don’t know | 61% (n=51)  27% (n=22)  5% (n=4)  2% (n=2)  5% (n=4) | |
| 4b. IF MORE THAN 1 CHILD WITH SCD: what type of SCD do your children have? (select all that apply) (N=18)  SS  SC  SBeta+ thalassemia  Sbeta0 thalassemia  I don’t know  No response given | 50% (n=9)  39% (n=7)  6% (n=1)  0% (n=0)  11% (n=2)  6% (n=1) | |
| 5. What is the race/ethnicity of child/children with SCD? (select all that apply)  African American or Black  African or Black  Caribbean or Creole  Hispanic or Latinx  White  Multiracial | 78% (n=79)  22% (n=22)  2% (n=2)  1% (n=1)  1% (n=1)  1% (n=1) | |
| 6. Does anyone else in your family have SCD?  Yes  No I don’t know | 25% (n=25)  68% (n=69)  7% (n=7) | |
| 7. Do you have any other children who live in the home with you?  Yes, 1 child  Yes, 2 children  Yes, 3 children  Yes, 4 children  Yes, 5 children  Yes, 6 or more children  No other children | 33% (n=33)  20% (n=20)  18% (n=18)  4% (n=4)  1% (n=1)  2% (n=2)  23% (n=23) | |
| 8. Does anyone else (other than your child/children) live in the home with you?  Yes  No | 40% (n=40)  60% (n=61) | |
| 9. Do you consider yourself the primary caregiver for anyone else (other than your child/children)?  Yes  No  No response given | 51% (n=52)  48% (n=48)  1% (n=1) | |
| 10. Is there anyone else that shares caregiving responsibilities with you for your child with SCD?  Yes  No | 61% (n=62)  39% (n=39) | |
| 11. Is there anyone else that shares caregiving responsibilities with you for anyone else in the home?  Yes  No | 45% (n=45)  55% (n=56) | |
| 12. On average, how long does it take you to get to Sickle Cell Comprehensive Clinic?  Less than 20 minutes  20-60 minutes  1-2 hours  Over 2 hours  No response given | 35% (n=35)  47% (n=47)  12% (n=12)  6% (n=6)  1% (n=1) | |
| 13. About how many miles are you from the Sickle Cell Comprehensive Clinic?  Less than 25 miles  25-50 miles  Over 50 miles | 65% (n=66)  20% (n=20)  15% (n=15) | |
| 14. Do you currently have health insurance?  Yes  No | 90% (n=91)  10% (n=10) | |
| 15. Does your child with SCD currently have health insurance?  Yes  No | 97% (n=98)  3% (n=3) | |
| 16a. IF CAREGIVER OR CHILD CURRENTLY HAS HEALTH INSURANCE: Have there ever been times when you or your child did not have health insurance, even if only for a short period of time? (N=100)  Yes  No | 38% (n=38)  62% (n=62) | |
| 16b. IF CAREGIVER OR CHILD DOES NOT CURRENTLY HAVE HEALTH INSURANCE: If you feel comfortable, can you share some of the reasons why you or your child with SCD have been unable to get health insurance? (N=12)  Too expensive to purchase  Unemployed  Other  Prefer not to answer | 8% (n=1)  42% (n=5)  42% (n=5)  8% (n=1) | |
| 17. Has insurance or the lack of insurance ever kept you from getting the care your child with SCD needed?  Yes  No | 13% (n=13)  87% (n=88) | |
| **Sickle Cell Experience** |  | |
| 18. When did you find out that your child has SCD?  At birth/newborn screening  Other | 84% (n=85)  16% (n=16) | |
| 19. If you are the birth parent, did you know you carried Sickle Cell Trait before your child was born?  Yes  No  I am not the birth parent | 53% (n=54)  38% (n=38)  9% (n=9) | |
| 20. How has your experience getting care for regular sickle cell visits been? Scale: 0-10 (N=99)  Mean  Median  Standard deviation | 2.36  2  2.40 | |
| 21. How often do you meet with your child’s sickle cell provider (doctor, nurse practitioner, etc.) in a year?  Once a year  2-3 times a year  4 or more times a year | 13% (n=13)  47% (n=47)  41% (n=41) | |
| 22. Does your child have other medical providers they see? (select all that apply)  Dentist  Eye doctor  Neurology  Neurosurgeon  Psychologist/Therapist/Mental Health Worker  My child does not see any other medical providers  Other  No response given | 73% (n=74)  35% (n=35)  19% (n=19)  8% (n=8)  12% (n=12)  3% (n=3)  35% (n=35)  2% (n=2) | |
| 23. Does your child have a primary care provider, for example a pediatrician or family doctor?  Yes  No | 90% (n=91)  10% (n=10) | |
| 24. How easy is it to schedule appointments with your PCP? Scale: 0-10 (N=98)  Mean  Median  Standard deviation | 2.22  2  2.39 | |
| 25. In total, how many medical appointments (does not include visits to the emergency room) does your child with SCD have in a year?  0-3 appointments  4-6 appointments  7 or more appointments | 45% (n=45)  35% (n=35)  21% (n=21) | |
| 26. Do you feel that having a child with SCD has impacted your day-to-day stress levels?  Yes  No  No response given | 41% (n=41)  58% (n=59)  1% (n=1) | |
| 27. Do you feel that having a child with SCD has impacted your levels of depression and/or anxiety?  Yes  No  No response given | 39% (n=39)  60% (n=61)  1% (n=1) | |
| 28. Do you worry about others (school, daycare, etc.) taking care of your child?  Yes  No  Sometimes | 28% (n=28)  37% (n=37)  36% (n=36) | |
| 29. Do you worry about others (school, daycare, etc.) not knowing enough about sickle cell disease?  Yes  No  Sometimes | 47% (n=47)  28% (n=28)  26% (n=26) | |
| 30. Do you worry about other healthcare providers (doctor, nurse, etc.) other than your sickle cell team taking care of your child?  Yes  No  Sometimes | 21% (n=21)  59% (n=60)  20% (n=20) | |
| 31. Do you worry about healthcare providers (doctor, nurse, etc.) other than your sickle cell team not knowing enough about sickle cell disease?  Yes  No  Sometimes  No response given | 35% (n=35)  49% (n=49)  16% (n=16)  1% (n=1) | |
| 32. Have you ever felt like your child did NOT receive the care they needed by an emergency room provider (doctor, nurse, etc.)?  Yes  No | 10% (n=10)  90% (n=91) | |
| 33. Have you ever felt like your child did NOT receive the care they needed while hospitalized?  Yes  No | 8% (n=8)  92% (n=93) | |
| 34. Have you ever felt like your child did NOT receive the care they needed in sickle cell clinic?  Yes  No | 2% (n=2)  98% (n=99) | |
| 35. The hospital sets up all appointments to be on the same day:  Always true  Sometimes true  Never true  No response given | 36% (n=36)  44% (n=44)  20% (n=20)  1% (n=1) | |
| 36. It is easy to access hospital staff (via phone, email, etc.):  Always true  Sometimes true  Never true  No response given | 54% (n=55)  44% (n=44)  1% (n=1)  1% (n=1) | |
| 37. It is easy to schedule appointments:  Always true  Sometimes true  Never true  No response given | 76% (n=77)  22% (n=22)  1% (n=1)  1% (n=1) | |
| 38. I am able to schedule appointments at times that are convenient for me:  Always true  Sometimes true  Never true  No response given | 66% (n=67)  32% (n=32)  0% (n=0)  2% (n=2) | |
| 39. The hospital has helped me when I had money problems related to my child’s care:  Yes  No  Not applicable  No response given | 17% (n=17)  33% (n=33)  50% (n=50)  1% (n=1) | |
| 40. The hospital has supported me in other ways (select all that apply):  Food  Mental health support  Transportation  Housing/rent  Utility bills  Not applicable/none  Other  No response given | 11% (n=11)  18% (n=18)  17% (n=17)  7% (n=7)  6% (n=6)  13% (n=13)  50% (n=50)  3% (n=3) | |
| 41. I have received support from:  Religious institution (Church, Masjid, Synagogue, etc.)  Local charity  Friends  Family  No applicable/none  No response given | 8% (n=8)  3% (n=3)  10% (n=10)  68% (n=69)  9% (n=9)  2% (n=2) | |
| **“Please answer how often the following items affect your ability to access medical care or attend medical appointments for your child/children with sickle cell disease.”** | |  |
| 42. Getting to the doctor’s office:  Almost never  Sometimes  Almost always  No response given | 52% (n=53)  27% (n=27)  20% (n=20)  1% (n=1) | |
| 43. Getting hold of the doctor’s office or clinic:  Almost never  Sometimes  Almost always  No response given | 54% (n=55)  27% (n=27)  18% (n=18)  1% (n=1) | |
| 44. Having to wait too many days for an appointment:  Almost never  Sometimes  Almost always  No response given | 72% (n=73)  23% (n=23)  4% (n=4)  1% (n=1) | |
| 45. Getting care after hours or on the weekends:  Almost never  Sometimes  Almost always  No response given | 58% (n=59)  30% (n=30)  11% (n=11)  1% (n=1) | |
| 46. Having to take care of household responsibilities:  Almost never  Sometimes  Almost always  No response given | 39% (n=39)  41% (n=41)  20% (n=20)  1% (n=1) | |
| 47. Having to take time off work:  Almost never  Sometimes  Almost always  No response given | 36% (n=36)  45% (n=45)  19% (n=19)  1% (n=1) | |
| 48. Having to wait too long in the waiting room:  Almost never  Sometimes  Almost always  No response given | 56% (n=57)  38% (n=38)  5% (n=5)  1% (n=1) | |
| 49. Meeting the needs of other family members:  Almost never  Sometimes  Almost always  No response given | 45% (n=45)  40% (n=40)  15% (n=15)  1% (n=1) | |
| 50. The cost of visits/healthcare:  Almost never  Sometimes  Almost always  No response given | 54% (n=55)  33% (n=33)  11% (n=11)  2% (n=2) | |
| 51. My child having to miss school:  Almost never  Sometimes  Almost always  No response given | 40% (n=40)  43% (n=43)  17% (n=17)  1% (n=1) | |
| 52. Number of visits to the office or clinic:  Almost never  Sometimes  Almost always  No response given | 51% (n=52)  38% (n=38)  10% (n=10)  1% (n=1) | |
| 53. Lack of resources within the hospital:  Almost never  Sometimes  Almost always  No response given | 75% (n=76)  22% (n=22)  2% (n=2)  1% (n=1) | |
| 54. Language barrier/lack of interpretation services:  Almost never  Sometimes  Almost always  No response given | 87% (n=88)  10% (n=10)  2% (n=2)  1% (n=1) | |
| 55. Not knowing what services you have access to:  Almost never  Sometimes  Almost always  No response given | 59% (n=60)  32% (n=32)  8% (n=8)  1% (n=1) | |
| 56. Do any of these barriers affect you or your child’s overall health and well-being?  Yes, my child’s health/well-being  Yes, my health/well-being  Both my child and I’s health/well-being  No  No response given | 6% (n=6)  3% (n=3)  11% (n=11)  78% (n=79)  2% (n=2) | |
| 57. Do any of these barriers affect you or your child’s overall mental and emotional health?  Yes, my child’s mental/emotional health  Yes, my mental/emotional health  Both my child and I’s mental/emotional health  No  No response given | 5% (n=5)  6% (n=6)  8% (n=8)  79% (n=80)  2% (n=2) | |
| 58. Have any of your healthcare providers or clinics discussed potential solutions to these barriers or provided you with resources?  Yes  No  No response given | 41% (n=41)  55% (n=56)  4% (n=4) | |
| **Telemedicine** |  | |
| 59. Have you ever participated in a telemedicine or virtual visit?  Yes  No  I don’t know  No response given | 40% (n=40)  46% (n=46)  11% (n=11)  4% (n=4) | |
| 60a. IF PREVIOUS PARTICIPATION IN TELEMEDICINE OR VIRTUAL VISIT: was this for a sickle cell visit? (N=40)  Yes  No | 78% (n=31)  23% (n=9) | |
| 60b. IF NONE OR UNKNOWN PREVIOUS PARTICIPATION IN TELEMEDICINE OR VIRTUAL VISITS: have you heard of telemedicine or virtual visits? (N=57)  Yes  No  I don’t know | 51% (n=29)  39% (n=22)  11% (n=6) | |
| 61a. IF PREVIOUS PARTICIPATION IN TELEMEDICINE OR VIRTUAL VISIT: what type of telemedicine have you participated in? (N=40)  You go to a local clinic and see your doctor on a video screen (Hub-and-spoke)  You are at home or work and see your doctor on your phone or laptop (Direct-to-consumer) | 8% (n=3)  93% (n=37) | |
| 62. Which of the following do you believe are positives about telemedicine/virtual visits? (select all that apply)  No transportation time or costs  Less time off work  Easier for my other caregiving responsibilities (finding childcare, etc.)  Convenient  Easier to access healthcare specialists who may be far away  Less chance of getting sick (COVID-19, flu, etc.)  None of the above  Other  No response given | 49% (n=49)  27% (n=27)  22% (n=22)  51% (n=52)  31% (n=31)  36% (n=36)  4% (n=4)  12% (n=12)  4% (n=4) | |
| 63. Which of the following do you believe are negatives about telemedicine/virtual visits? (select all that apply)  Difficulty with Wi-Fi  I do not have access to smartphone/tablet/laptop  No physical exam  Security concerns  Harder to build trust between provider and patient  None of the above  Other  No response given | 26% (n=26)  5% (n=5)  43% (n=43)  6% (n=6)  24% (n=24)  10% (n=10)  11% (n=11)  3% (n=3) | |
| 64. Would you be willing to participate in telemedicine or virtual care for your child’s sickle cell care?  Always  Sometimes  Never  No response given | 14% (n=14)  67% (n=68)  17% (n=17)  2% (n=2) | |
| 65. Would you be willing to participate in telemedicine or virtual care for your child’s other medical appointments?  Always  Sometimes  Never  No response given | 10% (n=10)  71% (n=72)  17% (n=17)  2% (n=2) | |
